# “Well, You Feel More Responsible When You’re Unsupervised”: Exploring Family Perspectives on Children’s Independent Mobility

**DOI:** 10.3390/children8030225

**Published:** 2021-03-15

**Authors:** Negin A. Riazi, Mariana Brussoni, Patricia Vertinsky, Guy Faulkner

**Affiliations:** 1 School of Kinesiology, The University of British Columbia, Vancouver, BC V6T 1Z4, Canada; patricia.vertinsky@ubc.ca (P.V.); guy.faulkner@ubc.ca (G.F.); 2 Department of Pediatrics, The University of British Columbia, Vancouver, BC V6H 3V4, Canada; mbrussoni@bcchr.ubc.ca; 3 School of Population and Public Health, The University of British Columbia, Vancouver, BC V6T 1Z3, Canada; 4 British Columbia Children’s Hospital Research Institute, Vancouver, BC V6H 3N1, Canada; 5 Centre for Hip Health and Mobility, Vancouver, BC V5Z 1M9, Canada

**Keywords:** built environment, communication, confidence, family, social cohesion, social connection, social norms

## Abstract

While children’s independent mobility (CIM) is associated with various benefits, there is evidence of a generational decline in CIM in westernized countries; therefore, it is helpful to understand how CIM is currently negotiated between children and their parents. The purpose of this study was to examine children’s and parents’ perspectives and negotiations of CIM within the family unit. Face-to-face interviews and walk-along interviews were conducted with parents (*n* = 44) and children (*n* = 22), respectively. Interviews were audio-recorded and transcribed verbatim, and a thematic analysis was conducted. Four key preconditions were identified that facilitated negotiation of CIM within family units, including (1) the influence of parents’ childhood experiences regarding their view of CIM (e.g., positive interpretations of childhood on parenting practices), (2) the role of children’s individual characteristics on their independent mobility (e.g., child’s confidence in their abilities), (3) family communication as a key coping strategy (parent–parent and parent–child communication), and (4) the influence of positive perceptions of the social environment on CIM. The findings suggest that CIM thrives when these conditions are present; as a result, it may be particularly helpful to develop policies and programs that support children’s skill training, explore strategies to support communication between parents and children, and build neighbourhood connections.

## 1. Introduction

Children’s independent mobility (CIM) (a child’s freedom to travel within their neighbourhood without supervision by adults) [1] is associated with benefits including greater physical activity [2], improved risk assessment, way-finding skills, self-confidence, and self-esteem [3]. However, challenges like unsupportive social norms, parental fear of societal judgement, and the potential involvement of social services exist for CIM [4,5]. For example, in 2017 one Vancouver father gained international media attention after being faulted by the Ministry of Children and Family Development for allowing his four children (ages 11, 9, 8, and 7 years at the time) to take public transit to school by themselves [6]. Even after the father spent two years training his children to take the bus to their school, the Ministry ruled that his children under 10 years of age could not be unsupervised on the bus [7]. While CIM research has increased, what is concerning is the generational decline in CIM levels [5,8]. This decline may consequently affect children’s physical activity levels as well as other social, cognitive, and developmental benefits gained from roaming the neighbourhood independently, such as better acquisition of environmental knowledge, spatial-mapping skills, interaction with peers, and enhancing a sense of community [9,10,11]. 

A variety of social–ecological correlates influence CIM, including individual factors (e.g., child’s age, gender), social environment factors (e.g., perceptions of stranger danger, traffic), and built environment factors (e.g., distance, destination availability) [3,12]. Previous quantitative research has identified many social–ecological correlates of CIM, but there is a need for more in-depth examination of these factors and exploration of their nuances. For instance, some studies showed boys and older children tended to have higher levels of IM than girls and younger children [12], while other studies showed the opposite [13,14]. Qualitative methodologies may be better suited to exploring these inconsistencies by focusing on the “contexts” and “experiences” of individuals and “emphasizing the voices of participants” [15] (p. 4), such as how other factors like children’s personalities and maturity may be influential for CIM, as these individual characteristics are hard to quantify especially in quantitative studies. 

Limited qualitative research has focused on exploration of families’ (e.g., children and their parents) perspectives on CIM. Brown and colleagues [16] examined children’s (11–12 years old) gender differences on their way to acquiring independence in two locations in southeast England. While boys had higher levels of CIM, girls gained similar levels by adjusting how they traveled (i.e., in groups). Additionally, Crawford and colleagues [17] conducted focus groups with 132 children (8–15 years old) and a small group of parents (*n* = 12) examining experiences and perceptions of CIM in metropolitan and regional areas in Victoria, Australia representing varying physical and community environments. The study highlighted that children reported a wider variety of safety concerns in comparison to parents, but also that children liked having independent mobility. Additionally, parental decision-making regarding their children’s level of independence was influenced by parents’ safety concerns (e.g., people- and traffic-related risks) [17]. Often, mothers’ perspectives are predominantly represented in CIM literature [18,19] emphasizing an underrepresentation of partners’ perspectives. For example, a multi-site primary school-based study examining correlates of CIM found that mothers (79%) were more likely to fill out the parent survey [20]. This may underscore an absence of research focusing on diverse family perspectives on CIM, the interactions within households, or how these households experience CIM. 

A recent study examined factors that facilitated or hindered children’s unsupervised outdoor play through children’s interviews and found two overarching themes relating to children’s sense of safety and perceptions of things to do in the neighbourhood [21]. While this study highlighted the child perspective, it also emphasised a need to examine CIM more comprehensively from multiple perspectives. Children exist within the family unit and are co-creators of that family environment; considering a single perspective, whether it be the child or parent, overlooks other important perspectives within that unit. It is important to consider multiple perspectives in the family unit to provide a comprehensive understanding of how CIM is negotiated within the family context. Although extant literature has emphasized parents’ gatekeeping role (e.g., children’s physical activity, outdoor play) [22], it is equally important to include children’s perspectives and participation in the “issues that affect them” [23]. As highlighted through family systems theory, interactions within family units are complex, connected, and often interdependent [24]. Importantly, little CIM research focuses on the context and precursors of negotiation of CIM within the family unit.

The purpose of this study was to examine child and parental perspectives and negotiations of CIM within the family unit. Two questions guided our investigation: What are different family members’ (parents and child) perspectives of CIM in relation to attitudes and approaches to CIM? How do parents and children negotiate CIM? This examination is important in advancing our understanding of the influence of individual-, social-, and built-level factors on CIM and how families negotiate CIM and may provide important insight for the development of policy and practice interventions to increase CIM. 

## 2. Materials and Methods

### 2.1. State of Play Study

This qualitative study was embedded in a larger cross-sectional study (State of Play) conducted in the Greater Metro Vancouver area, Canada. The State of Play study explored the socio-ecological perspectives on children’s outdoor play and CIM. Families (children 10–13 years old and their parents) were recruited from three distinct neighbourhoods in the Metro Vancouver area, including Vancouver’s Grandview–Woodland, North Vancouver’s Lonsdale, and Richmond’s Steveston. These neighbourhoods were selected for their variation in urbanisation, population density, ethnic make-ups, and number of children (see [25] for further detail). For example, the neighbourhood types were considered urban (Vancouver), urban–suburban mix (North Vancouver), and suburban (Richmond) [25]. This is reflected by population density with the population per km^2^ being 5492.6, 4465.1, and 1534.1 for Vancouver, North Vancouver, and Richmond, respectively. The population under 15 years old was lowest in Vancouver (11.2%) compared to North Vancouver (13.4%) and Richmond (13.7%) [25]. Families were recruited through word-of-mouth, social media (e.g., targeted Facebook ads,), community centres and libraries, and local parent listservs. Criterion-based sampling was used to select participants. Inclusion criteria included living within one of the target communities, reading, speaking, and understanding English. Additionally, children had to be able to participate in physical activities at the time of study and were able to roam either independently (or with friends) at least within their yard and/or driveway. Thirty-five families were interviewed in each of the neighbourhoods (*n* = 105 child interviews; *n* = 127 parent interviews). Ethics approval was obtained from the institutional Research Ethics Boards at The University of British Columbia and BC Children’s and Women’s Hospital (H15-02190).

### 2.2. Participants

Twenty-two families were purposefully sampled from the larger study sample to select information-rich cases to shed light on the questions being investigated [26]. Families were included if both parents completed interviews and the child completed a walk-along interview. Sixty-six total interviews met the inclusion criteria (22 child interviews; 44 parent interviews) and were included in the analysis. The included family units were spread across the three neighbourhoods (Vancouver, *n* = 10; North Vancouver, *n* = 6, Richmond, *n* = 6). Participants largely self-identified as White (*n* = 49; 74.2%) or Asian (*n* = 13; 19.7%), as a woman (*n* = 24), or man (*n* = 20), as heterosexual (*n* = 20 families), or same sex couples (*n* = 2 families). Child participants self-identified as girls (*n* = 9), boys (*n* = 12), and non-binary (*n* = 1) (see Table 1 and Table 2 for participant demographics). Close to half (45.5%) of parents fell in the 41–45 age range, and child mean age was 11.5 years old. Most children (82%) had levels of independent mobility ≥4.6 according to parent-reported scores (Table 2 reports the average independent mobility score from both parents’ reported scores; 1–6 scale). Each parent and child received $50 and $100, respectively, for participating in the study.

### 2.3. Data Collection

Face-to-face interviews were conducted with parents, and walk-along interviews were conducted with children. Interviews provide a way for participants to share their experiences in “rich and detailed ways” (p. 108) and acknowledge that participants’ perspectives and interpretations on those experiences are shaped by social factors [27]. During the walk-along interview, the researcher accompanied the participant on “outings in their familiar environments” (p. 264), in this case, the child’s neighbourhood [28]. The walk-along interview method is flexible, helps build rapport, and reduces power dynamics between the researcher and participant [28]. Parents were asked about differences in their independent mobility approach, their childhoods, general philosophy around independent play, and questions related to CIM barriers (e.g., fears, concerns) and facilitators (e.g., neighbourhood connectedness, safety). Each parent also reported their child’s independent mobility by scoring on a scale from 1 (my child is not allowed out alone) to 6 (my child is allowed out more than a 15-min walk from home). Children were asked about time playing outside, whether they were unsupervised/independent, the range they could travel, as well as barriers and facilitators of CIM. Additionally, perceptions of individual, social, and built environment factors were explored (see Supplementary Material S1 for interview guides).

Interviews were conducted by the principal investigator (MB), research coordinator, and a team of research assistants (RAs) (*n* = 6), including the first author (NR). All members of the interview team were trained in qualitative interviewing by the research coordinator prior to leading interviews. Semi-structured interview guides (see Supplementary Material S1) were used for their flexibility, consistency, and comparability, since participants were interviewed by several interviewers (RAs) [29]. Parent interviews lasted approximately 60 minutes, children’s walk-along interviews lasted 45–60 min, and all interviews were conducted between April 2016 and February 2018. Most of the data collection occurred during the months that children were in school during autumn and spring (September to October; March to June).

### 2.4. Data Analysis and Quality

All interviews were audio-recorded, transcribed verbatim by a local professional transcriber or RA from the State of Play research team, and checked for accuracy by a RA. This study adopted a social constructivist approach, which acknowledges that individuals develop “subjective meanings” from their experiences, and these meanings are “varied and multiple” [30] (p. 24), and the researcher attempts to interpret the meanings others have about the world [30]. Data from the parents’ and children’s interviews were analysed through reflexive inductive thematic analysis [31,32]. QSR International’s NVivo 12 Software was used to organise and code the data. Each interview in a family unit was coded before moving on to the next family unit. Family units within the same neighbourhood were coded before moving on to the next neighbourhood. Codes were identified for each family unit, across each neighbourhood, then across the full dataset, and themes were developed that reflected the patterns generated across the dataset. 

A variety of steps were taken during data collection and analysis to address the multiple perspectives (i.e., both parents and child) in the data. Interviews were conducted separately with each participant to address power relations, the potential silencing of the voice of the child and/or parents influencing children’s answers or each other’s answers [33]. Children, however, could have a sibling, parent, or friend attend the walk-along interview if that helped them feel more comfortable. The first author worked to provide equal consideration of children’s and parents’ accounts through several strategies to maintain reflexivity, including keeping analytical notes during the analysis (e.g., regarding reasoning, questions, and emotional reactions to interviews), as well as engaging with critical friends throughout the analysis [33,34]. Critical friends included the co-authors of this paper and a colleague who have expertise in CIM, physical activity, outdoor play, and qualitative methods; they acted as sounding-boards to stimulate discussion, provide feedback on conclusions and data interpretations, and encourage the researcher’s reflexive acknowledgement of results and perspectives in the research process [34]. 

## 3. Results

The findings are presented in this section. First, an overview of children and parents’ common barriers to independent mobility is provided. The findings are then presented as four overarching themes that resonated with the 22 families. Pseudonyms are followed by participant age, neighbourhood (Vancouver = V; North Vancouver = NV; Richmond = R), and family (e.g., F1, F2, etc.). 

Most children felt “comfortable” and “good” about being independently mobile in their respective neighbourhoods. As Ryan (13, V, F2) described,

“…on Sundays I go for bike rides with my friend, and we don’t actually quite have a plan on where we’re going, we just sort of end up somewhere. Which I think is really cool…And that’s where I found out about most of my favourite spots”.

While a few children preferred being in the company of a friend, being home instead of outside, or were sometimes more comfortable with an adult around, overall, most children valued and enjoyed their independent mobility. Angela (12, R, F21) spoke about her mother’s expectations,

“Like my mom like she’s like, ‘I’m not here to supervise you so you can go and do whatever you want as long as you’re not getting in trouble.’ So I’ll just go out, as long as we’re not like being like irresponsible or like getting into doing bad things, she’s fine with it”.

Families discussed a variety of concerns focused mostly on the social environment with some concern about children’s individual characteristics. Concerns were often consistent within the family unit. The most cited concern was traffic (e.g., cars, dangerous street crossings, reckless drivers). Amongst families in Vancouver and North Vancouver, the second most discussed concern was related to drug use—people smoking, doing or dealing drugs, and appearance of needles or syringes on the ground. Additionally, parents and children discussed concerns about homeless people in these neighbourhoods and/or individuals who were in an altered mental state due to poor mental health or intoxication. Across all neighbourhoods, parents raised concerns about children’s individual characteristics (e.g., lack of awareness, ability to evaluate situations). Additionally, other concerns were raised by families including stranger danger, “sketchy people” (Caleb, 13 R, F19), and worries about kidnapping or abduction. Broadly, there was consistency in the perspectives of children and parents regarding these concerns and potential barriers to CIM.

Despite families’ concerns, most children exhibited moderate to high levels of independent mobility. In exploring families’ perspectives and negotiations of CIM, four major themes were identified that provide insight into key preconditions leading to the negotiation of independent mobility between parents and children. These included (1) the influence of parents’ childhood on their views of CIM, (2) the role of children’s characteristics on their independent mobility, (3) communication as a key coping strategy for families, and (4) the influence of perceptions of the social environment on CIM. 

### 3.1. “It’s a Great Sense of Freedom”: The Role of Parents’ Own Childhood Experiences on Children’s Independent Mobility

Most parents painted a stark picture between their childhoods and those of children today. Parents spoke about having “free reign” and the ability to go “anywhere and everywhere” (Nelson, 47, V, F6) and being “…allowed a lot more freedom than I think kids are today” (Lauren, 45, V, F6). Parents consistently referred to their childhood independent mobility freedoms. For example, Meghan (42, R, F17) remarked that as a child, she “walked to kindergarten by [herself] and came home”. Parents’ own childhood experiences, the value they placed on independent mobility, and the consequent parent-reported benefits may have influenced their CIM levels. Parents wanted their children to experience the same freedoms they themselves had as children. Lily (53, R, F21), explained that independent exploration allowed “…you [to] learn to be more independent because you get to make mistakes and learn something…” Parents valued the unstructured time, outdoor play, and lessons learned from and through those independent experiences. 

Although parents spoke favourably of the freedoms afforded to them as children, they emphasized shifts in societal norms. Barbara (52, V, F7) spoke about how she had a “happy childhood” with lots of “unstructured play” and believed that it was “great for kids to be playing outside. But I think that the world that [son] is growing up in…is very different from the one that I grew up in”. One of these differences was the absence of technology. Nora (52, V, F10) explained that even though,

“…nobody had cellphone or pagers or anything…it was just great to go out and have an adventure and catch tadpoles in jars and do all that sort of exploring and make up our own games…and we did it for years, and we loved it and we’d go exploring all over the neighbourhood”.

Even without technology, parents were not restricted from exploring their environment. During parents’ childhoods, independent mobility took the form of travel and unstructured free play allowing opportunities to explore, engage their imaginations, and be immersed in the outdoors. 

Several families discussed changing norms around acceptable parenting practices and judgement regarding their parenting. One father explained that while he and his partner try to encourage their children to “be independent and to be active and to play…but at the same time, …what we would let them do and not do…it’s now modulated by what is accepted in our society today and it’s probably less than what we had when I was a child myself” (Adam, 48, NV, F11). Some parents were reluctant in allowing their children certain freedoms (e.g., independent travel and play) based on how society views those activities (e.g., judgment for allowing child to roam freely). This sentiment was echoed by other parents who wanted their children to experience a childhood like their own,

“I do feel some societal pressure at times to hover a little bit…there is a certain level of I think expectation in the media that you’re gonna do everything you can to make sure that your child is healthy and successful and, god forbid you let your child play in the dirt because they’ll get salmonella or something like that”

(Scott, 42, V, F3)

This societal shift from “come back when the streetlights come on” (Nora, 52, V, F10) to an expectation of constant supervision was a topic frequently discussed, reiterating the dissonance between parents’ and children’s childhood experiences. 

Additionally, parents discussed a generational rise in fears about what potential harm could befall children. One father, Richard (44, V, F5), said,

“my mom and dad like to remind me that they weren’t scared when I went off…we lived about two or three kilometers from the school I went to, and they weren’t scared of me getting abducted or anything like that”.

A few parents admitted they would not necessarily allow the freedoms they had for their child because of concern about children “playing outside like that” (Edward, 40, R, F22). Additionally, a couple of families raised concern over judgement from others and societal expectations. Lauren admitted, “…there’s a lot of judgement about what your kids are allowed to do” and she explained an incident where another parent from school raised concern over her children walking home from school by themselves. Lauren admitted that “…from then on I felt much more cautious about letting my kids…roam on their own than I think I would’ve if that hadn’t happened…” 

Although parents pointed to stark generational differences, there was consistent agreement across most interviews that parents valued their childhood independent mobility freedoms and wanted to recreate that for their children. Margaret (48, V, F4) remembered, “It was a pretty good childhood and we want basically the same kind of thing for our kids…” Her partner Daniel (50, V, F4) explained,

“I mean that’s the lifestyle that we like and we also want our kids to have…we don’t want to be helicopter parents and be supervising all the time…it’s important for them [children] to you know, develop the responsibility on their own and self-reliance on their own”.

Parents often referred to their own childhoods when making decisions about their CIM. As Dave (42, V, F2) mentioned,

“I use that to really inform myself on a daily basis when they ask for more freedom on things … but we’re really hoping and starting to see that he could very well be emulating that kind of freedom that we both cherished”.

Most parents agreed with their partners on their CIM philosophies, comfort levels, and benefits independent mobility could provide for children. However, some differences existed in approaches or perspectives on CIM, specifically regarding level of concern between parents. In one family, Lily (53, R, F21) explained that her partner, Oliver (53, R, F21), was “…probably a lot less concerned. We say if [Oliver] is in charge just know that they’re not being, well, supervised…” while Oliver acknowledged that, “Yeah, we differ in our parenting styles…I basically let [daughter] do more than her mother lets her get away with”. Interestingly, their daughter Angela (12, R, F21) felt that her parents were “both totally fine with me being outside, being unsupervised”. It may be that the child perceived the combined decisions of her parents’ decision-making and negotiations. Other parents negotiated these differences by implementing their own rules when the child was with them. Ella (45, NV, F11) described that, “When he [Adam] is in charge, it’s his rules. But if I’m in charge, it’s my rule”. Her partner, Adam (48, F11), explained that they differed, but “not in a huge fashion and I tend to be a little more permissive than she is”. While differences in their parenting styles were acknowledged, these differences may not have been sufficient to meaningfully affect CIM.

### 3.2. “A Very Trustworthy Kid”: Children’s Individual Characteristics and Their Independent Mobility

Children’s and parents’ perceptions of children’s individual characteristics (e.g., child age, confidence) helped shape CIM. Although children’s age was discussed as a factor for independent mobility, other characteristics were more dominant in conversation. Parents who felt their child had their “act together” (Karen, 45, V, F5) (e.g., aware of surroundings, ability to deal with unexpected situations, able to navigate neighbourhood, trustworthy) were less worried about perceived dangers (e.g., traffic, stranger danger, abduction) since their child had the abilities to address those dangers. 

One family in Vancouver spoke about negotiating their concerns for their son, Jake’s safety. The father explained, “…he’s a very trustworthy kid. He’s smart. I’m not worried about him. Like, he’s got good street smarts…” (Richard, 44, V, F5). The mother, Karen (45, V, F5), echoed these sentiments,

“I think he’s a really responsible kid… he makes really good decisions. I think he evaluates situations really well… I guess I could start getting concerned about all the things that could go wrong, but I just…that would just take up all your time…I think he’s smart and capable and can think on his feet, and I think he’s fine”.

Jake’s (11, V, F5) perspective also aligned with his parents’ assessments when he explained, “And I just know it’s [smoking and drugs] bad to do and I just stay away from those people, and I know where is a good place for me to be and where’s not”.

Parents affirmed they worried about their children being independently mobile but also recognized a need to have confidence in their children’s ability to safely navigate the neighbourhood. Bill (49, NV, F16) explained, “Yeah. I mean—you’re always on edge, you’re a parent,” but went on to say:

“I mean you kind of got to trust that, you got that confidence in them that they’re going to be okay… if you go around living your life, worrying that they’re never going to come home because he’s going to get hit by a car…You’ll never let them out”.

Other parents worried about their children’s individual characteristics such as personality or maturity. Claire (41, NV, F13) explained that she worried about George (12, NV, F13), because he was “very trusting”. “George assures me no [he is not too trusting], but that’s one concern…he may not always understand what people’s motives, and intentions…a big worry…”. The perception that children did not have adequate “problem-solving skills” (John, 48, V, F1), the ability to deal with situations on their own, or being naïve or too trusting were other characteristics discussed amongst families. For example, Cayden’s (10, V, F7) assessment of himself aligned with his parents’ Barbara (52, V, F7) and Angie’s (45, V, F7) assessments in which the whole family unit acknowledged the child’s anxiety playing a role in limiting his independent mobility.

Cayden: “…if I don’t know when I’m going to meet them [parents], then I start panicking because…I have high anxiety”.

*Researcher: Do you think having high anxiety has any effect on the way you play outside?*

Cayden: “Well I mean, I’m less adventurous”.

Barbara: “He gets anxious about some things…”

Angie: “And he’s also an anxious personality as well…but also personality-wise he’s quite introverted and also very much in his own world… I don’t know if he would necessarily pick up on sort of social cues that would help him read a situation that might be dangerous”.

However, Julie (10, V, F1), questioned why her parents Katherine (46, V, F1) and John (48, V, F1) worried about her. Julie explained that, “for some reason I’m not allowed to walk to the bus stop, but I can still take the bus by myself…they [parents] still don’t like it when I go there”. Her parents, however, raised concerns about their daughter’s ability to solve unexpected problems plus assess situations and people. Katherine worried about “[Julie’s] decision-making, problem solving ability and just how she evaluates situations, safety situations… so I’m like, you know, you just need to be able to show me that you identify who…where the issues are more before I let you go”. Her partner, John, agreed that he was waiting to see “[Julie’s] problem solving skills” develop so that he could “be more comfortable with her going further out or walking to places by herself”. Most parents who believed their children were confident, cautious, and had good problem-solving abilities positively influenced parents’ confidence in their child’s capacity to navigate their neighbourhood environment safely. 

### 3.3. “A Little More Peace of Mind”: Communication as a Coping Strategy

Both parents and children discussed communication as an important facilitator of CIM. Communication encompassed four topics: the logistics of communication (e.g., with whom, when), communication within the family unit, safety-related communication, and technology as a communication tool. There was a general expectation that children would communicate certain details when being independently mobile. For example, asking for permission, identifying how long, or until what time they would be gone, and where they were headed. Tracy (13, V, F10) described her independent mobility experience:

“My parents aren’t really that strict because they know I’m safe and they know the neighbourhood pretty well…. There isn’t really a limit. I always tell my parents where I’m going, and how long I’ll be gone for, and when I’ll be back. They’re always just like, ‘Have your phone with you, and always answer calls or texts right away. Don’t ignore them.’”

As long as Tracy communicated with her parent(s), she had an extensive roaming range. Her mother, Nora (52, V, F10), echoed her sentiment explaining that “…as long as I know where she’s going, and if she’s with someone if it’s an area I don’t know…. So if she’s with a group of people, sure that’s fine”. Albert (49, V, F10) said that when Tracy first started taking the bus,

“she was a little nervous about it at first, but we were pretty encouraging and cool about it. And it didn’t take much. Like first day, my wife took the bus with her, and like the next day Nora was like ‘do you want me to that with you again?’ and Tracy was like ‘no, I got it.’ And that was fine with us”.

Communication also facilitated negotiation of independent mobility within the family unit. Parents often discussed boundaries of CIM amongst themselves before discussing with their child. Dave (42, V, F2) described discussions between him and his partner about Ryan’s (13, V, F2) expanding independent mobility:

“Not as a daily conversation, but as Ryan’s world expands, we definitely you know, ‘so is it okay for him to hang out in the mall and stuff,’ ‘yeah okay sure’… but I think when we talk about how we feel about him doing things outside of the home, I think we both are starting and ending in a place of let’s find a way to be eye-to-eye on this so we’re a unified unit when we talk to him about it, so we’re not giving him mixed messages on it”.

Differences in parents’ comfort were often negotiated through discussion and a mutual decision was reached. A few parents, however, acknowledged they allowed either more or less independent mobility if they were the ones making the decision rather than their partner. 

Communication in the family unit helped build parents’ confidence in children’s ability to navigate the environment safely. Ryan (13, V, F2) explained that, “As long as I let my parents know, pretty much as far as I want” and his mother, Lisa (41, V, F2), seemed quite at ease about Ryan’s roam range, “Pretty far…so like he goes to Metrotown on his own, he goes to Downtown on his own, he visits friends”. Interestingly, some parents commented that they were ready to give their child more independent mobility; however, their child had not broached the topic yet. As Dave (42, V, F2) explained:

“…[Ryan] hasn’t asked me to go anywhere yet where I’ve gone ‘oh that’s too far.’ …long as, if I know he can get there, if I just ask him how he can get there then I’m confident he can get back…”

Occasionally, children were unsure the extent of their roaming range as this was never explicitly discussed with their parents. When Casey (12, NV, F15) was asked how far she could travel, she responded, “I’m not really sure. I guess I just have to like ask them [parents]”. Discussions between parent and child about CIM were a precursor to facilitating the expansion of independent mobility. 

Safety discussions within the family unit were key to addressing safety-related concerns. These discussions focused on salient concerns within each family, including awareness, drug safety, cycling safety, traffic safety, homelessness, and people in altered mental states. Kathleen (49, NV, F16) explained that her son, James (11, NV, F16), had “been told over the years what we expect of him and how to look after himself and be safe”. These safety discussions helped parents convey important, need-to-know information about the neighbourhood and helped prepare children on how to safely roam the neighbourhood. 

Cell phone technology arose as an important resource. More than half of children had a cell phone, although the phone’s calling and texting capabilities and data plan varied. Some children only had an iPad/iPod with messaging and calling capabilities via Wi-Fi. Parents whose children had a communication device (e.g., cell phone, iPod) felt it was a tool to open a line of communication. Daniel (50, V, F4) explained that his son’s cell phone was “more of a safety valve or resource”. “Well I just know like… if he gets lost or in a situation that he’s gonna have a hard time handling, he can always call us”. A few parents of children without cell phones discussed being “more comfortable” (Marc, 43, R, F20) if their child had a way to communicate with them. Some children checked-in using a friend’s phone, an adult’s phone, used a Wi-Fi hotspot to text via another device, or had a more limited roaming range. Most of the children with a cell phone felt that having a phone provided a sense of safety and many parents agreed. Kathleen (49, NV, F16) explained that:

“…having the phone to communicate with [James] (11, NV, F16) allowed me to relax…he can communicate back the way ‘Oh if you’re running late, can I go to …so and so’s house now and I’ll meet you back at home?’”

Additionally, cell phones either facilitated a greater independent mobility range or parents confirmed that independent mobility would increase once the child had a phone. As Trevor (12, V, F9) explained, “I think having a phone has had an impact on how far I feel like I can go. Because then I can always find my way with Google Maps”. While Trever felt his parents afforded him a wide range of independent mobility, having a phone allowed him and his parents greater comfort in traveling alone. However, some parents preferred their children to be older (preferably high school) before receiving a cell phone because of concerns around increased screen time and exposure to social media. Lily (53, R, F21), whose daughter did not have a cell phone, talked about the dangers of all “those social media things” because “…I don’t think that they [children] know how to govern themselves with it or what they do with that information that they get out of it”. Instead, Lily’s daughter Angela (12, R, F21) typically traveled “in pairs” with a peer and communicated “where [she’s] going” and the “timeframe” with a parent. Family discussions of safety and readiness were prominent for negotiating CIM and access to communication devices helped ease concerns.

### 3.4. “You Look Out for People”: The Social Environment and Children’s Independent Mobility

Families’ perceptions of the social environment were prominent in shaping CIM. Participants felt safe in their respective neighbourhoods; this arose from presence of a “kid culture” Lisa (41, V, F2) (i.e., other children in the neighbourhood), familiarity with the neighbours and neighbourhood, and a sense of informal social control. As one mother, Lisa (41, V, F2), explained, “there is a very live kid culture in this area; it’s why people flock here when they have families because they can feel it right?” Another family explained benefits of having other children in the neighbourhood including interaction with peers. Allen (42, NV, F15) commented, “We have great neighbours around so then the kids play out like 3 or 4 girls and they have some girls from across the street as well”. Additionally, Laura (40, NV, F15) explained why it was important for the family to live in a neighbourhood that had children, “I always felt like if you live in the community where other kids are…they [children] have a chance to interact and be out…”. Their daughter, Casey (12, NV, F15), also highlighted the availability of peers in her neighbourhood, “I usually hang out with my friends a lot. And I have a lot of friends nearby”. 

Families emphasised that children in the neighbourhood helped create connections between neighbours. As Lauren (45, V, F6) explained, “the people I know the best are people who my kids have played with”, and Nelson (47, V, F6) affirmed: “The neighbour on that side, our eldest daughter babysits their kids. [Lydia] babysits the cat across the street…. We had a block party which was really good, we’ve had that for a number of years”. On her walk-along interview, Lydia (11, V, F6) also indicated friends’ and neighbours’ homes in the neighbourhood, “…that blue house over there. [Pebbles], is a cat that…[I] cat-sit. That’s my house. That is my friend’s house, just over there”. This “kid culture” in the neighbourhood helped create opportunities for children to be independently mobile, play, and travel with their peers. Monica (57, V, F9) echoed the importance of having a neighbourhood community,

“And we live in a nice neighbourhood with people who are also at that school and who are very community-minded…. We’re not really interested in like a big house or a lot of stuff. For us, it’s more important to have a good community…”

Furthermore, familiarity with the neighbours, neighbourhood, and a sense of informal social control helped both parents and children feel safe and often led to greater independent mobility. For example, one North Vancouver family explained their familiarity with their neighbours:

Brigette (11, NV, F11): “I know pretty much like all the people on my block and they’re all really friendly”.

Ella (45, NV, F11): “I talked to other people and they said that no other neighbourhood had people coming to knock at their door to play…. I guess we were really lucky we had amazing neighbours”.

Adam (48, NV, F11): “We knew the neighbours, we knew that there were other children, we knew that there were people always in parks you know, walking their dogs and so people we know would help a child…”.

Most families felt the presence of people in the neighbourhood (*eyes on the street*) and informal social control (where other members of the neighbourhood took collective responsibility for children) [35] promoted a sense of safety as well as friendliness. As Freddy (11, R, F17) explained, he liked having people out and about in his neighbourhood, “…like my brother fell the other day and then like minutes later all the adults were around to help him so it’s like the perfect amount of supervision because nobody is there yet everyone is there, you know”. His mother, Meghan (42, R, F17), spoke about the community in her neighbourhood, where,

“It’s sort of a group mentality of parenting that everybody sort of knows where the other kids are… I don’t even know where my kids are right now, they’re out there somewhere…. Somebody will call me if they need me or they’ll come…. I think that that’s really invaluable”.

Many participants commented on how they felt comfortable letting their child go out independently and/or children felt at ease outside because families knew others in the neighbourhood would help if a child should need it.

Knowing neighbours would help a child allowed many participants to feel comfortable about CIM. Additionally, parents’ concerns (e.g., traffic) were typically for outside the vicinity of their immediate neighbourhood. As Lisa (41, V, F2) clarified, her worries increased when her child went outside the neighbourhood,

“But once they get out of this neighbourhood, and I worry sometimes, like we have conversations like ‘you have to be careful when you’re walking in the suburbs like you have to watch because people aren’t paying attention in their cars.’”

Overall, families acknowledged that their neighbourhoods felt safe, neighbours looked out for each other, and people would lend a helping hand if it was needed.

## 4. Discussion

This study contributes to the growing CIM literature by identifying several pre-conditions that may be important for facilitating negotiations of CIM within family units. Most parents had positive interpretations of their childhood independent mobility. Although recalling past memories may blur the “exact factual details” of the past (i.e., rose-coloured glasses), the emotional ties, whether positive or negative, can often be accepted [36] (p. 4) as meaningful. Positive views of their childhood independent mobility experience may have translated to motivations and attitudes that influenced their own children’s upbringing (e.g., aim to provide similar freedoms to their children). While parents encouraged CIM, they also played a role in defining its limits via parent–parent discussion about children’s travel range, destinations they could travel to, safety discussions pertinent to the neighbourhood, and parent(s)–child discussions around these topics. These findings highlight that the linked nature of family members [24] and the filtering of parents’ perspectives to children may consequently impact children’s freedoms, including the timing and range of CIM [21]. While positive childhood recollections were common in this study, it may be helpful for future initiatives to encourage parents to reflect on their childhoods, their independent mobility, and benefits and/or skills they gained, and reframe the risks they may perceive for their children. For example, one recent online tool helps parents reframe the risk of children’s outdoor play through self-reflection and experiential learning tasks [37].

Additionally, findings emphasised concerns around societal judgement and shifting social norms. These included culturally constructed ideals of parenting practices, fear of judgement, declining freedoms like CIM, and shifts toward more organised activities for children [13,19,38,39]. The Vancouver father’s case described earlier [6] highlights current social norms, parenting practices, and potential judgment. Generational shifts in norms have further impacted constructions of good parenting and children’s geographies. Pynn and colleagues [40] examined generational parenting ideals and active free play and reported that changing expectations of parental involvement, influence of news media on perceptions of safety, and worries of judgment on social media were all related to shifting parenting ideals. Over time, children’s space and geographies have changed, in that children “do not play outside that often; they have less freedom of movement; and they have a smaller territory” [41] (p. 289). A study examining children’s time use in the United Kingdom over 40 years found a significant decrease in children’s outdoor play [42]. Future research and initiatives targeting CIM should acknowledge the influence of societal norms on parenting and, consequently, the trickle-down effect on CIM.

Various individual characteristics are associated with CIM including child age, gender, and skills [43,44,45]. Our findings shed light on the complexity of children’s individual characteristics and highlight both children’s and parents’ perceptions of the child’s maturity, skills, and abilities as crucial to negotiating CIM. If a child was confident, could “think on their feet” (Karen, 45, V, F5), had “street smarts” (Richard, 44, V, F5), and displayed good decision-making skills, it helped alleviate parental concerns, regardless of their child’s age or gender. Additionally, children’s confidence in their own abilities to navigate the environment typically led them to feel more comfortable traveling and playing in their neighbourhoods. Parental uncertainty regarding their child’s abilities hindered CIM, more so than consideration of child’s gender and age. This may explain the inconsistent associations in the literature between these individual characteristics and CIM. Other research shows that parental attitude toward independent travel is related to children’s characteristics and also to parental factors like the need to protect the child [44]. One prevalent view in Western society is that children are seen as “vulnerable” and in need of protection, and often good parenting is tied in with parental risk assessment or a culture of “protectionism” [46,47]. Therefore, parents wanting to promote their child’s independent mobility (e.g., case of Vancouver father) need to balance allowing their children freedom to explore and practice good parenting by protecting their children from potential danger. 

Perceptions of the social environment were an important focus amongst families, which is consistent with research demonstrating that social environment-level correlates may by particularly salient for CIM, including parents’ perceptions of safety and social cohesion [4,44,48]. In this sample, feelings of community and safety played an important role in facilitating CIM, especially when families engaged in safety discussions addressing concerns (e.g., drugs, traffic, homelessness). Although families spoke about concerns, these concerns were largely moderated by positive perceptions of the social environment, particularly feelings of community, knowing that people in the neighbourhood looked out for each other, and familiarity with neighbours. Speculatively, this may explain why objective measures of the built environment are not necessarily associated with CIM. Feelings of community and safety are a necessary facilitating precondition for CIM to occur irrespective of objectively-measured environmental attributes [49].

Communication within family units was crucial for negotiation of CIM. Communication allowed children to display their confidence, skills, or lack thereof, and helped parents assess whether children were ready for greater independent mobility. Interestingly, cell phones (or other messaging-capable devices) were seen as a positive tool [20,50], a tool for communication between family members [51], and provided parents with a sense of security and social control [52]. Children’s independent travel is more common once a child turns 13 or 14 [53], but for younger children, parents wield more influence over the distance or destinations their children travel to independently. Building communication capacity between parents and children may be crucial for increasing CIM; for instance, a cell phone (or other communication device like an iPod) or knowledge that a child’s school will call parents if a child does not arrive at school may be ways in which children could be afforded independent mobility earlier. 

Multi-level and multi-sectoral approaches should be considered for addressing CIM [54]. At the individual-level, it is important to help children develop the skills (e.g., traffic safety; communication), competence, and confidence to navigate their neighbourhood safely, and to support parents and partners (e.g., schools) to support children. At the social-level, interventions should target building of neighbourhood connections and social capital to facilitate CIM; for example, investing in initiatives that help promote neighbourhood connection (e.g., neighborhood grants for the organisation of bloc parties) or initiatives like Play Streets, which have been shown to increase community and provide safe places for play and physical activity [55]. To target the built environment level, implementation of safe cross walks and protected cycling lanes could objectively make environments safer [56] and could influence parents’ and children’s safety perceptions of the environment. Finally, policies that address broader macro-level factors such as substance use harm reduction initiatives, social welfare, or policies supporting CIM may influence CIM through mitigating some common concerns raised by parents and children. 

### Strengths and Limitations

Strengths of this study included a relatively large sample size (*n* = 66) in three distinct neighbourhoods varying in urbanization. Examination of the family unit through walk-along and face-to-face interviews highlighted the complexities of CIM negotiations including parent–parent discussions, which could consequently impact parent(s)–child discussions, as well as drawing attention to varying comfort-levels of family members and how these were addressed within the family. However, the sample focused on two-parent households (*n* = 22) with most participants being of higher socioeconomic status. We acknowledge the privileged nature of our sample. Additionally, families in this study appreciated the importance of CIM and were motivated to support CIM. An exploration of perspectives of single-parent households, families varying in socioeconomic status, and living in rural locations or disadvantaged neighbourhoods, and children with low levels of independent mobility would be informative, as there may be different perspectives that were not captured in this study. Recent research has highlighted how living in a disadvantaged neighbourhood may impact parents’ defensive behaviours (e.g., limiting locations of play, requiring supervision), which limits outdoor play and independent mobility for children and adolescents [57]. These defensive behaviours may arise as a result of parental perceptions of the neighbourhood environment, including perceptions of higher crime or traffic insecurity, poor social capital, or lack of physical activity resources (e.g., parks, green space) [57]. It would also be beneficial to view CIM prospectively to examine changes in independent mobility over time, rather than relying on recollections, to reduce recall bias.

## 5. Conclusions

With decreased CIM levels in many westernized countries, it is important to identify key conditions that may help facilitate CIM. The findings suggest that CIM flourishes where and when the conditions are conducive, including (1) individual characteristics of the child, confidence in their own abilities, as well as parents’ confidence in their child’s abilities to safely navigate the neighbourhood; (2) parent–parent communication (e.g., discussing the range appropriate for the child to travel) and parent–child communication (e.g., check-ins, demonstration of skills); (3) the influence of positive interpretations of parents’ own childhood on parenting practices; and (4) positive perceptions of the neighbourhood social environment. Findings also emphasized the need for multi-level and multi-sectoral initiatives that can target the diverse social–ecological factors that may impact CIM (e.g., built environment, social environment). Whether these conditions can be modified through intervention or how CIM can be supported when these conditions do not exist will require future research attention. 

## Figures and Tables

**Table 1:** Parental demographic characteristics.

Demographics	Parent	
	*n*	%
**Age range**		
36–40	4	9.1
41–45	20	45.5
46–50	13	29.5
51–55	6	13.6
56+	1	2.3
**Race/ethnicity**		
White	33	75.0
Asian	9	20.5
Prefer not to answer	2	4.5
**Marital status**		
Common law	27	61.4
Divorced	16	36.4
Married	1	2.3
**Employment status**		
Employed for wages	30	68.2
Homemaker	2	4.5
Self-employed	9	20.5
Both self-employed and part-time employed	1	2.3
Student	1	2.3
Working part-time	1	2.3
**Education**		
Post grad	15	34.1
University	19	43.2
Some university	2	4.5
College	7	15.9
Some college	1	2.3
**Household income**		
Unable to work	1	2.3
<$29,999	1	2.3
$50,000–$69,999	1	2.3
$70,000–$89,999	5	11.4
$90,000–$109,999	6	13.6
$110,000 to $129,999	9	20.5
≥$130,000	21	47.7

**Table 2:** Child demographic characteristics.

Demographics	Child	
	*n*	%
**Age range**		
10	4	18.2
11	8	36.4
12	5	22.7
13	5	22.7
**Grade in school**		
4th	3	13.6
5th	2	9.1
6th	8	36.4
7th	4	18.2
8th	5	22.7
**Ethnicity**		
White	16	72.7
Asian	3	13.6
Mixed	3	13.6
**Independent mobility level**	(range 1–6)	
1–2.5	2	9.1
2.6–3.5	1	4.5
3.6–4.5	1	4.5
4.6–5.5	9	40.9
5.6–6	9	40.9

## Data Availability

The data presented in this study are available on request from the corresponding author. The data are not publicly available due to privacy.

## Supplementary Materials

The following are available online at https://www.mdpi.com/2227-9067/8/3/225/s1, S1: Interview guides.
