# Supplementary material for: “Well, You Feel More Responsible When You’re Unsupervised”: Exploring Family Perspectives on Children’s Independent Mobility
Source: Children (Basel). 2021 Mar 15;8(3):225. doi: 10.3390/children8030225 (PMC7998357; doi:10.3390/children8030225)
Supplement: Supplementary file 1 [file children-08-00225-s001.pdf]

S1: Interview Guides

Interview Guide – Children

Map-Elicitation Interview Questions:

PART 1 objective: Go over the daily maps together and compare them against the daily surveys. This is to clarify the periods of independent mobility. ***Record the interview***. [In the beginning of the recording, state your name, the date of the interview, the participant code and the segment of the interview]

- How did you get from A to B? With who? How long did it take?
- What did you do there? With who? For how long?

PART 2 objective: To understand children's perspectives towards their outdoor environment and independent mobility, and what they want in their neighborhood.

General background:

- What are some of your favourite *fun things** to do after school and on the weekend, and how much time do you spend doing these? (Query indoors vs. outdoors activities, with who).
- In a typical week in spring and summer, how much time do you spend doing fun things? With who? (your parents, siblings, friends) (If necessary, query weekday vs. weekend).
- Where do you usually go to hang out or play outside? What do you do? With who?

**Fun thing* means any kind of activities that <u>you</u> find enjoyable and amusing. This may include but not limited to playing at the playground, drawing or painting, playing a sport together, taking a hike, working a puzzle etc.

Children's unsupervised outdoor play:

- In a typical week in spring and summer, how much time do you spend outside playing? How much of this time is without your parent(s) or another adult around?
- How far from home are you allowed to go without your parent(s) or another adult around? Do you ever go past that point?
- What kinds of things are you allowed to do without your parent(s) or another adult around?
- How do you feel about playing outside without your parent(s) or another adult around?
- What would make you feel more comfortable going outside without your parents or another adults around?
- Do boys and girls play outside differently?

Children's perceptions of their neighborhood and built environments:

- Are there things you'd like to do in your neighbourhood that you don't get to do? What are some of the things that make it difficult for you to do these things?
- Do you feel safe playing outside in your neighbourhood? What concerns you the most?
- Do you find your neighbors friendly?

Go-Along Interview Questions:

Objective: Ask children to take you on a tour around their neighborhood, and take pictures of places they want to tell you about. ***Record the interview and wear a GPS watch for the tour.*** *[In the beginning of the recording, state your name, the date of the interview, the participant code and the segment of the interview]*

- Where: where do you usually hang out? Your favorite or least favorite place to hang out? Places that are meaningful to you? Any of your friends live close to you?
- Who: Who do you usually play with here (friends, siblings, parents, by yourself)?
- What: What do you usually do when you hang out here? Favorite thing to do when hanging out here? Things that you would like to do but you can't?
- Why: Why do you like/dislike this place? What is special about this place? How does it make you feel when you play here?

- ➢ Give $80 honorarium to the child and get his/her signature on the corresponding honorarium receipt.
- ➢ Debriefing
- ➢ Complete research field notes part III – child interview.

Interview Guide – Parents

Objective: This is to better understand parents' perspectives towards children's outdoor play (independent or supervised) and their perceptions of built environment – as well as how different factors (e.g., individual and social factors) impact children's outdoor play. ***Record the interview***. [In the beginning of the recording, state your name, the date of the interview, the participant code]

General background:

- What are some of your favourite things to do with [child]? (If necessary, query indoors vs. outdoors activities).
- In a typical week, how much time do you spend doing *fun things** with [child]? (If necessary, query weekday vs. weekend, summer vs. winter).
- Are there things you'd like to do with [child] that you don't have a chance to do now? What are some of the things that make it difficult for you to do these things with [child]?

**Fun thing* means any kind of activities that your child finds enjoyable and amusing. This may include but not limited to playing at the playground, drawing or painting, playing a sport together, taking a hike, working a puzzle etc.

Children's unsupervised outdoor play:

- What does 'unsupervised play' mean to you?
- In a typical week in spring and summer, how much time does [child] spend outside playing after school hours? On the weekend? How much of this time is *unsupervised*?
- Where do you think [child] is when [child] is playing outside unsupervised? What do you think [child] is doing?
- How far from home is [child] allowed to go without supervision?
- What kinds of things is [child] allowed to do without supervision?
- How do you feel about [child] playing outside without you or another adult around?
- What things concern you about [child] going out unsupervised?
- At what age did you let your child go out unsupervised?
- Would you feel differently if [child] was a [opposite gender]?

Parents' perceptions of their neighborhood and built environments:

- What things would make you more comfortable in allowing [child] to go out unsupervised? (If necessary, query attitudes toward neighbourhood, social circumstances, built environment).

- Do you feel connected to your neighbours? (i.e. would someone be there to help your child while they were playing in your neighbourhood?)
- Do you feel safe in your neighborhood? Do you feel safe for [child] in your neighborhood?
- What types of crime concern you for [child]'s safety? Do you feel there is a lot of crime in your neighborhood?
